# Prevalence and correlates of depression among chronic kidney disease patients in Taiwan

**DOI:** 10.1186/1471-2369-14-78

**Published:** 2013-04-04

**Authors:** Hsin-Hung Chiang, Hanoch Livneh, Mei-Ling Yen, Tsai-Chung Li, Tzung-Yi Tsai

**Affiliations:** 1 Department of Nursing, Buddhist Dalin Tzu Chi General Hospital, Chiayi, Taiwan; 2 Rehabilitation Counseling Program, Portland State University, Portland, USA; 3 Graduate Institute of Biostatistics, College of Public Health, China Medical University, Taichung, Taiwan; 4 Department of Healthcare Administration, College of Health Science, Asia University, Taichung, Taiwan; 5 Department of Medical Research, Buddhist Dalin Tzu Chi General Hospital, Chiayi, Taiwan; 6 Department of Nursing, Tzu Chi College of Technology, Hualien, Taiwan; 7 Department of Environmental and Occupational Health, College of Medicine, National Cheng Kung University, Tainan, Taiwan

**Keywords:** Depression, Chronic kidney disease, Prevalence, Taiwan

## Abstract

**Background:**

Chronic kidney disease (CKD) is a progressive disease that causes a permanent impairment of renal function and premature mortality. The associated prognosis may result in serious psychological distress to the affected individual. However, there are limited data on the psychological correlates, and in particular depression, in Chinese CKD patients. This study aimed to examine the prevalence of depression, as well as the influence of other psychosocial factors on depression, among Taiwanese CKD patients.

**Methods:**

We used a cross-sectional research design to recruit 270 CKD patients who were not undergoing dialysis treatment at a hospital in southern Taiwan during 2011. The structured questionnaire used in this study gathered information on respondent demographic and disease characteristics, and information obtained from the Taiwanese Depression Questionnaire. Factors associated with depression were examined by a multiple logistic regression analysis.

**Results:**

The crude and age-standardized prevalence of depression were 22.6% and 20.6%, respectively. Those who had sleep disturbances, reported having no religious beliefs, followed no regular exercise regimen, and were diagnosed with stage III or above CKD demonstrated a significantly higher risk of depression.

**Conclusion:**

Our findings are beneficial to healthcare providers, as they identify both the prevalence of depression and several of its correlates. By identifying CKD patients with a higher risk of depression, healthcare providers may be better able to ensure the provision of appropriate rehabilitation to this population.

## Background

Chronic kidney disease (CKD) is becoming a major public health concern. The worldwide prevalence of end-stage renal disease (ESRD) is expected to continue to rise at an annual rate of 7% and exceed 2 million by 2010, which will cost about 1.1 trillion dollars in medical expenditures [1]. Recently, based on a report by the United States Renal Data System published in 2007, Taiwan had the highest incidence and prevalence rate of ESRD patients globally, with 415 and 2288 per million populations, respectively [2]. The soaring number of patients with renal disease resulted in a heavy economic burden to the healthcare system. According to a report by the Taiwan National Department of Health published in 2009, the number of ESRD patients only accounted for 0.2% of all beneficiaries, but consumed 32.8 billion NTD (New Taiwan Dollar), which is approximately 7.1% of the entire Medicare budget, and surpasses the medical expenditures on cancer (18.3 billion NTD) [3].

CKD not only causes enormous economic losses, but also triggers major challenges in regards to health. Wen and colleague [4] found that CKD patients had an 83% higher rate of all-cause mortality and were twice as likely to die from cardiovascular disease. Due to the irreversible nature and poorer prognostic outcomes, psychiatric disorders, in particular depression, are frequently common among patients with renal disease [5-7]. One study found that ESRD patients have a nearly 4-fold higher risk for depression than the general population [8]. One cause of concern was that depression not only elevated the length of hospitalization by 30% [9], but also more than doubled the likelihood of mortality in ESRD patients with depression compared to those with ESRD only [8]. However, in contrast to available data on ESRD, there is a limited amount of information regarding depression among patients with earlier stages of CKD. Recently, a study demonstrated that depression in CKD patients, who were not receiving dialysis treatment, was linked to a 86% higher risk of adverse events including death, early dialysis initiation or hospitalization risk [10], suggesting that depression is a matter of great concern during the routine care of CKD patients.

Although some studies on depression and its contributing factors among CKD patients have been conducted, the majority of the studies were performed in Western countries [10-12]. Influenced by the Asian conservative culture, Chinese often regard depression as a taboo and are, thus, highly reluctant to mention this openly to others [13]. Thus, a review of the literature indicates that most reports on Taiwanese CKD patients have focused on the effects of medical treatment [14], self-care behaviours [15], and possible pathogenic factors [16]. Conversely, research findings on depression among Chinese CKD patients are scarce. This study aimed to examine the prevalence of depression and related correlates among Taiwanese CKD patients, with the hope that findings may suggest appropriate psychological intervention strategies for Asian CKD patients.

## Methods

### Study design and population

This cross-sectional study adopted a purposive participant recruitment and was carried out at a hospital in southern Taiwan to recruit CKD patients who were not undergoing dialysis treatment from either outpatient or inpatient departments between January and August of 2011. The inclusion criteria were as follows: (i) being 20 years of age or older; (ii) having no cognitive impairments and being able to express opinion in either Mandarin or Taiwanese; (iii) being aware of their CKD and not yet undergoing dialysis treatment; and (iv) being able to consent to a blood test on the survey date. The sample size needed for the study was estimated by using the methodology of Hsieh et al. [17], where α was set to 0.05, power was set to 0.8, the event rate at mean of X was set to 0.03 [4], and the odds ratio of 3.56 per unit increase in X [18]. It was determined, based on these psychometrics, that a sample of at least 167 patients was required for data analysis.

### Instruments

A set of measures was used for data collection. These included the Taiwanese Depression Questionnaire (TDQ), and an additional questionnaire that obtained information on demographic and disease characteristics.

The TDQ, developed by Lee et al. [19], was chosen, as it was specifically created with the Asian culture in mind. It consists of 18 items, each addresses those symptoms that have been experienced over the past week using a scale of 0 (absence of symptoms) to 3 (presence of symptoms almost every day) scores. Total scale scores, therefore, can theoretically range from 0 (low depression) to 54 (high depression). After using the Structured Clinical Interview for DSM Disorders (SCID) manual as the gold standard, the TDQ was shown to have good concurrent validity, with the area under the Received Operating Characteristic (ROC) curve being 0.92. The TDQ also performed optimally, at a cutoff value of 19 or greater, in detecting the presence of depressive symptoms, whether for patients with kidney disease or for the general population [19-21]. In regards to its reliability, the TDQ has previously demonstrated good internal consistency among different groups of subjects, with a Cronbach’s α ranging between 0.90 and 0.92 [13,20,21]. The Cronbach’s α derived from the present data yielded a coefficient of 0.89.

The second part of the questionnaire contained information on demographic and disease characteristics, developed from previous literature and clinical experiences. Information on patients’ demographic data included gender, age, marital status, educational level, job status, living status, religious beliefs, and certain lifestyle factors, such as smoking, alcohol consumption, exercise habits, and sleep disturbance. Those who answered “currently” or “yes/past” to smoking were classified as smokers. Alcohol consumption was stratified into two groups based on whether they had consumed alcohol at least two times per week. Those who exercised 3, or more, days per week were classified as having regular exercise habits. As for information about sleep disturbances, to be congruent with the timeframe used for data collection, it was obtained by a single question that asked participants how often they have awakened suddenly from sleep during the past week. Those who have been awakened more than twice per week were classified as having sleeping problems. The disease characteristics included the following: chronic disease (i.e. at least one of the following: diabetes mellitus, hypertension, heart disease, or a stroke), CKD stage, disease duration of CKD, hemoglobin level, creatinine level, and albumin level. All disease characteristics were obtained from reviews of the patients’ charts.

### Data collection

This study was approved by the Institutional Review Board of Buddhist Dalin Tzu Chi General Hospital. The researchers explained the purpose of the study and its procedures to all of the patients. Informed consent was obtained after the patients understood and agreed to participate in the study. During the completion of the questionnaires, the researchers were available to answer any inquiries regarding the questionnaires. For illiterate patients, the researchers read them the questionnaires and recorded their answers. The questionnaires were returned without any identifying personal information. Patients were assured of complete confidentiality with respect to all of the obtained data. Patients were given the option to withdraw from the study at any time without any penalty.

### Statistical analysis

Descriptive and inferential statistical analyses were conducted in accordance with the study aims and the nature of variables. For descriptive analysis, subjects were grouped according to the presence of depression (i.e. cut off score of ≥19). Differences between groups on the various study variables were expressed in terms of means and the standard deviation (SD) or in percentage. Aside from age, disease duration and blood biochemistry markers that were expressed in means and SD, categorical variables were expressed as percentages. For inferential analysis, a *t*-test or chi-square test were used to explore the relationship between the study’s variables and depression. Multiple logistic regression analysis was further used to determine the various correlates and their adjusted odds ratios (AOR), in predicting the existence of depression. All analyses were conducted with SPSS version 12.0 (SPSS, Inc., Chicago, IL). A p < 0.05 was considered statistically significant.

## Results

### Demographic data and disease characteristics of CKD patients

A total of 270 CKD patients were recruited during the period of data collection. Of these, 61 individuals met the criteria for depression based on the TDQ total score of ≥19, which translates to a crude prevalence of 22.6% (61/270). After adjusting for age, based on the percentages of the 2000 World Standard Population, the age-standardized depression prevalence was found to be 20.6%. Thus, it was estimated that about one out of five CKD patients met the criteria for depression in our study.

In the present study, the mean age of the subjects was 64.5 years (SD =12.13), and most of the subjects were male (61.1%), married (93.0%), unemployed (73.0%), and cohabitating (91.1%). Additionally, most subjects reported having religious beliefs (84.4%), had a low level of education (74.8%), experienced sleep disturbances (63.0%), regularly engaged in exercise (64.1%), and had low level of alcohol consumption (94.1%). Nearly 60% of the patients were non-smokers. Furthermore, the mean disease duration of CKD was 3.53 years, and most patients presented with a CKD Stage IV or above (60.7%), as well as other chronic diseases (65.6%). The mean levels of albumin, hemoglobin, and creatinine were 3.51 g/dl, 11.08 g/dl, and 5.05 mg/dl, respectively (Table 1).

**Table 1:** **Demographic and disease characteristics of participants (*n*** **= 270)**

**Variables**	***M *( *SD *)**	***n *(%)**
**Demographic data**		
Age (years)	64.5 ± 12.13	
Gender		
Male		165(61.1)
Female		105(38.9)
Marital status		
Single		19(7.0)
Married (or divorced)		251(93.0)
Educational level		
Low (< 12th grade)		202(74.8)
High (≥ 12th grade)		68(25.2)
Job		
Employed		73(27.0)
Unemployed		197(73.0)
Living status		
Living alone		24(8.9)
Cohabitating		246(91.1)
Religious beliefs		
Yes		228(84.4)
No		42(15.6)
Sleep disturbance		
Yes		170(63.0)
No		100(37.0)
Alcohol drinking		
Low(≤1 times/week)		254(94.1)
High(≥2 times/week)		16(5.9)
Cigarette smoking		
Yes		111(41.1)
No		159(58.9)
Regular exercise		
Yes		173(64.1)
No		97(35.9)
**Disease characteristics**		
Disease duration (years)	3.53 ± 2.85	
CKD stage		
Stage III and below		106(39.3)
Stage IVand above		164(60.7)
Other Chronic diseases		
≤1		93(34.4)
≥2		177(65.6)
Hemoglobin (g/dl)	11.08 ± 2.03	
Creatine(mg/dl)	5.05 ± 1.81	
Albumin(g/dl)	3.51 ± 0.31	

### Correlations among demographic data, disease characteristics, and depression

Table 2 presented the demographic and disease characteristics among CKD patients who met criteria for depression and for those who did not. The results from the univatiate analysis revealed that CKD patients who were depressed were also more likely to be female (p = 0.03), single (p = 0.04), living alone (p = 0.02), reported no religious beliefs (p = 0.01), experienced sleep disturbance (p < 0.01), and did not engage in regular exercise (p = 0.03). Moreover, those with CKD stage IV or above tended to be more depressed (p = 0.02).

**Table 2:** **Relationship between demographic data, disease characteristics and depression risk (*n*** **= 270)**

**Variables**	**Depress (n = 61)**	**Non-depress (n = 209)**	***t/χ***^***2***^	***p***
**Demographic data**				
Age (Mean ± SD) (years)	65.70 ± 12.37	63.87 ± 14.78	0.95	0.34
Gender				
Male	30(49.2)	135(64.6)	4.72	0.03
Female	31(50.8)	74(35.4)		
Marital status				
Single	8(13.1)	11(5.3)	4.45	0.04
Married (or divorced)	53(86.9)	198(94.7)		
Educational level				
Low (<12th grade)	45(73.8)	157(75.1)	0.05	0.83
High (≥ 12th grade)	16(26.2)	52(24.9)		
Job				
Employed	11(82.0)	62(29.7)	3.24	0.07
Unemployed	50(18.0)	147(70.3)		
Living status				
Living alone	10(16.4)	14(6.7)	5.48	0.02
Cohabitating	51(83.6)	195(93.3)		
Religious beliefs				
Yes	45(73.8)	183(87.6)	6.83	0.01
No	16(26.2)	26(12.4)		
Sleep disturbance				
Yes	48(78.7)	122(41.6)	8.36	<0.01
No	13(21.3)	87(58.4)		
Alcohol drinking				
Low(≤1 times/week)	55(90.2)	199(95.2)	2.16	0.14
High(≥2 times/week)	6(9.8)	10(4.8)		
Cigarette smoking				
Yes	21(34.4)	90(43.1)	1.46	0.23
No	40(65.6)	119(56.9)		
Regular exercise				
Yes	32(52.5)	142(67.9)	4.94	0.03
No	29(47.5)	67(32.1)		
**Disease characteristics**				
Disease duration (Mean ± SD) (years)	4.04 ± 3.26	3.38 ± 2.70	-1.44	0.15
CKD stage				
Stage III and below	16(26.2)	90(43.1)	5.61	0.02
Stage IVand above	45(73.8)	119(56.9)		
Other Chronic diseases				
≤1	16(26.2)	77(36.8)	2.36	0.13
≥2	45(73.8)	132(63.2)		
Hemoglobin (g/dl)	10.71 ± 2.03	11.18 ± 2.02	1.60	0.11
Creatine(mg/dl)	5.40 ± 3.82	4.95 ± 4.28	-0.5	0.60
Albumin(g/dl)	3.50 ± 0.62	3.51 ± 0.48	0.04	0.97

### Predictor of depression in CKD patients

Multiple logistic regression analysis indicated that religious beliefs, sleep disturbances, exercise habits, and disease stage were significantly related to depression in these CKD patients. Compared to patients with religious beliefs, those with no religious beliefs had a 2.56-fold higher risk of depression (95% confidence interval (CI): 1.24-5.27). The risk for depression among patients with sleep disturbances was higher than for those with no sleep disturbance (AOR: 3.01; 95% CI: 1.37-6.62). CKD patients who did not engage in exercise had a significantly higher AOR for depression (AOR: 2.12; 95% CI: 1.14-3.96). Those diagnosed with CKD stage IV or higher were more than twice as likely to have depression (AOR: 2.14; 95% CI: 1.04-4.40) (Table 3).

**Table 3:** Risk factors for depression by multiple logistic regression

**Variables**	**Crude OR (95% CI)**	**Adjusted OR (95% CI)**
Gender		
Male	1	1
Female	1.88 (1.06-3.36)	1.64 (0.85-3.17)
Marital status		
Single	2.72 (1.04-7.09)	1.09 (0.25-4.80)
Married (or divorced)	1	1
Living status		
Living alone	2.73 (1.15-6.49)	3.07 (0.81-11.62)
Cohabitating	1	1
Religious beliefs		
Yes	1	1
No	2.50 (1.24-5.05)	2.56 (1.24-5.27)^**^
Sleep disturbance		
Yes	2.63 (1.35-5.15)	3.01 (1.37-6.62)^*^
No	1	1
Regular exercise		
Yes	1	1
No	2.24 (1.25-4.01)	2.12 (1.14-3.96)^*^
CKD stage		
Stage III and below	1	1
Stage IVand above	2.13 (1.13-4.01)	2.14 (1.04-4.40)^*^

^*^p < .05; ^**^p < .01.

## Discussion

To the best of our knowledge, there are no studies that have reported the prevalence of depression, along with its influencing factors, among Chinese CKD patients. This study found that the crude and age-adjusted prevalence of depression were 22.6% and 20.6%, respectively, revealing that one out of five CKD patients met the criteria for depressive disorders. This finding corraborates the 20% to 26% prevalence of depression found among renal disease patients who had received dialysis treatment [10-12]. It was inferred that CKD gradually induces fatigue, nausea, and loss of energy [22]. Furthermore, the changes in body image and inconvenience resulting from subsequent dialysis treatment may also heighten the level of depression in CKD patients [23]. Despite the fact that depression is increasingly prevalent among CKD patients, its detection and management are still not recognized in routine care [12]. Most importantly, a large number of Asian individuals view depression as a taboo and do not openly seek regular psychotherapy [24]. Therefore, healthcare providers must be cognizant of the existence of depression among CKD patients and inform them by standard care procedures to avoid possible embarrassment and discomfort when implementing early screening to identify and refer high risk cases [11,25].

With respect to the correlates of depression, the results of the multiple logistic analysis indicated that patients’ sex, age and marital status were not associated with depression among CKD patients, echoing previous findings [11,12,21]. The failure to observe any link between patients’ age and depression may also be partly due to the restricted age range of the participants and their older age (M = 64.5 years). Additionally, we found that living alone was not associated with an increased risk of depression. Despite the lack of comparable studies, the relationship between one’s living status and depression approached borderline significance. Further studies are still warranted to clarify the relationship between them. In regards to religious beliefs, patients with no religious beliefs were 2.5 times more likely to experience depression than those who reported having religious beliefs. It is possible that patients with religious beliefs had the opportunity to associate with other people and this experience may enhance psychological support, provide comfort through religious and spiritual counseling, and help relieve psychological pressures [26]. Our findings, however, differed from those reported by Chen et al. [21]. This inconsistency may be associated with the differences in the study participants (i.e. all subjects were hemodialysis patients in Chen et al. study) and statistical analysis methods (Chen et al. relied solely on univariate analysis). Using only univariate analysis to assess the relationship between religion and depression, instead of adopting a more comprehensive multivariate model that controls for potentially intervening covariates, may not accurately reflect the relationship between religion and depression.

This study also revealed that those with sleep disorders were three times more likely to have depression than those with no sleep problems. Despite the paucity of findings on the relationships between sleep disorders and depression among CKD patients, this finding is consistent with the theory that insomnia was related to the risk of depression in the general population [27]. From a neurobiological perspective, the arousal system of individuals who experience insomnia was more active than in those of the general population. This phenomenon would cause altered corticothalamic activities, such as higher concentrations of adrenocorticotropin (ACTH) and cortisol, which might further predispose them to develop psychiatric disorders [27,28]. Therefore, it could be suggested that periodic assessment of sleep disorders in CKD patients could be of utmost clinical importance to reduce the risk of depression.

Individuals who did not exercise had higher odds of developing depression than those who did, echoing a previous study [11]. Two reasons may account for this result. First, those who exercise regularly are able to strengthen their social network, thereby having more resources with which to face the impact of the disease. Second, a former study had reported that exercise could increase the concentration of certain monoamine neurotransmitters, such as serotonin, norepinephrine and dopamine [29], which may stimulate the brain to produce endorphins that help the individual feel happier and more relaxed [30]. However, Johansen et al. [31] found that over 60% of healthcare providers do not assess the exercise patterns or recommend appropriate exercise programs for patients with renal disease. Healthcare providers should design a personalized exercise program based on the patient’s level of physical functioning, including activities such as walking, tai-chi, yoga, or swimming, to delay the deterioration of renal function and further relieve stress associated with the disease [32]. Furthermore, these exercises were also recommended to be integrated into the home-based or dialysis-based training programs which may be beneficial in increasing the motivation levels of individuals with CKD to engage in exercise, thus decreasing the risk of depression [33].

The findings of our study also show that the more advanced the stage of kidney disease, the more likely the patient is to demonstrate a depressive mood. This finding differs from a report of Hedayati et al. [12]. It could be speculated that these conflicting findings are due to differences in the measurement tools and classifications used. For example, Hedayati et al. [12] used the Mini International Neuropsychiatric Interview Tool to examine the prevalence of depressive episodes, which was distinct from our scale (TDQ). Furthermore, the study by Hedayati et al. classified CKD by four stages (from stage II to stage V), which was also different from the classification system used in our study. Nevertheless, as it relates to disease stage, patients with more advanced stages of kidney disease typically experience a large number of symptoms [6]. The dread associated with entering dialysis treatment and the anxiety over one’s changing health status may further aggravate depression [34]. Therefore, it is imperative for healthcare providers to periodically evaluate the emotional status of CKD patients with more advanced stages (i.e. stage IV or above).

The results of this study should be interpreted with the following limitations in mind. First, the study subjects were recruited from a single hospital, which may limit the generalizability of the findings and, therefore, these findings cannot represent the depressive symptoms of all Chinese CKD patients, and certainly not of non-Chinese populations. This limitation, however, is not unique to our study. By their very nature, most studies are limited by such factors as subjects’ ethnicity, geographical location, nationality and the nature of the medical data available (e.g., levels of severity, functionality, symptomatology, duration of condition). Future studies should be conducted in a variety of regions of the country to examine if the findings are replicated within different demographic and geographic groups. Nonetheless, we calculated a required sample size analysis to ensure statistical power before embarking on the study, and thus the sample size used in this study may be considered satisfactory for exploring the factors associated with depression in CKD patients. Second, since this study used a cross-sectional design, we cannot infer causality from our findings. A longitudinal research design is needed to examine any causal relationships among the factors assessed in this study, such as pro-inflammatory cytokines. Third, the self-reported depressive symptoms yielded by the TDQ may differ from those observed by a trained physician, so the present findings should be interpreted with caution. Fourth, the sleep disturbance was only examined with a single-item question and therefore its psychometric characteristic may be suspect and, accordingly, must be interpreted cautiously. Despite these methodological concerns, to our knowledge, this was the first study to review the epidemiology of depression among Chinese CKD patients who were not treated by dialysis, a fact which can be useful in developing an early therapeutic regimen for Asian CKD patients.

## Conclusion

Modern medical technology can effectively extend the survival rate of patients with chronic diseases. However, the physical and mental distress that results from disease symptoms and their treatment may induce negative mood in the patient. In the present study, we found that the crude and age-standardized prevalence of depression among CKD patients were 22.6% and 20.6%, respectively. Patients with no religious beliefs, who did not regularly exercise, who had sleep disorders, and who were diagnosed with CKD stage IVor above, appeared to have a higher risk of developing depression. Healthcare providers should consider these findings as a reference for designing a standard rehabilitation program for CKD patients. Ensuring that mental health services are available to patients with CKD may be important first step to help them better psychologically adapt to their disease and possibly as important as improving the survival rate of patients with this chronic and life threatening disease.

## Abbreviations

CKD: Chronic kidney disease; ESRD: End-stage renal disease; NTD: New Taiwan dollar; TDQ: Taiwanese depression questionnaire; SCID: Structured clinical interview for DSM disorders; ROC: Received operating characteristic; SD: Standard deviation; AOR: Adjusted odds ratio; CI: Confidence interval; ACTH: Adrenocorticotropin

## Competing interests

The authors declare that they have no competing interests.

## Authors’ contributions

HHC provided administrative support and participated in providing comments on the manuscript drafts. HL contributed to the interpretation of data and providing comments on the final draft of the manuscript. MLY was responsible for data collection. TCL provided the statistical expertise and comments on the manuscript drafts. TYT was responsible for the study conception, design, data analysis, and drafting of the work. All authors read and approved the final manuscript.

## Pre-publication history

The pre-publication history for this paper can be accessed here:

http://www.biomedcentral.com/1471-2369/14/78/prepub
